# Altered natal dispersal at the range periphery: The role of behavior, resources, and maternal condition

**DOI:** 10.1002/ece3.2612

**Published:** 2016-11-30

**Authors:** Melissa J. Merrick, John L. Koprowski

**Affiliations:** ^1^ School of Natural Resources and the EnvironmentWildlife Conservation and ManagementUniversity of ArizonaTucsonAZUSA

**Keywords:** behavioral phenotype, condition‐dependent dispersal, maternal effects, peripheral population, phenotype‐dependent dispersal, *Tamiasciurus hudsonicus grahamensis*

## Abstract

Natal dispersal outcomes are an interplay between environmental conditions and individual phenotypes. Peripheral, isolated populations may experience altered environmental conditions and natal dispersal patterns that differ from populations in contiguous landscapes. We document nonphilopatric, sex‐biased natal dispersal in an endangered small mammal, the Mt. Graham red squirrel (*Tamiasciurus hudsonicus grahamensis*), restricted to a single mountain. Other North American red squirrel populations are shown to have sex‐unbiased, philopatric natal dispersal. We ask what environmental and intrinsic factors may be driving this atypical natal dispersal pattern. We test for the influence of proximate factors and ultimate drivers of natal dispersal: habitat fragmentation, local population density, individual behavior traits, inbreeding avoidance, competition for mates, and competition for resources, allowing us to better understand altered natal dispersal patterns at the periphery of a species’ range. A juvenile squirrel's body condition and its mother's mass in spring (a reflection of her intrinsic quality and territory quality) contribute to individual behavioral tendencies for movement and exploration. Resources, behavior, and body condition have the strongest influence on natal dispersal distance, but affect males and females differently. Male natal dispersal distance is positively influenced by its mother's spring body mass and individual tendency for movement; female natal dispersal distance is negatively influenced by its mother's spring body mass and positively influenced by individual tendency for movement. An apparent feedback between environmental variables and subsequent juvenile behavioral state contributes to an altered natal dispersal pattern in a peripheral population, highlighting the importance of studying ecological processes at the both range center and periphery of species’ distributions.

## 1 Introduction

Natal dispersal is a key process promoting gene flow, population viability, and species persistence in the face of rapid environmental change (Dieckmann, O'Hara, & Weisser, 1999; Gaines & McClenaghan, 1980). Natal dispersal distance, particularly long‐distance movement, is critical to predict a population's capacity to maintain gene flow, metapopulation dynamics, and colonize new areas (Sutherland, Harestad, Price, & Lertzman, 2000) and may be important in peripheral populations where habitat is patchy and gene flow is constrained. Dispersal outcomes are the product of interplay between extrinsic and intrinsic proximate factors (Clobert, Le Galliard, Cote, Meylan, & Massot, 2009), including site‐specific variation in density and conspecific sex ratios (Gaines & McClenaghan, 1980; Matthysen, 2005), availability and predictability of resources (Bowler & Benton, 2005; Le Galliard, Rémy, Ims, & Lambin, 2012), landscape patchiness (Matthysen, Adriaensen, & Dhondt, 1995), and phenotypic differences such as body size and condition (Debeffe et al., 2012), and systematic interindividual behavior differences, or personalities (Bowler & Benton, 2005; Cote, Clobert, Brodin, Fogarty, & Sih, 2010; Debeffe et al., 2013; Dingemanse, Both, Van Noordwijk, Rutten, & Drent, 2003; Duckworth, 2008), that may vary among populations. The decision to disperse, how far individuals disperse, and variation therein, while important, are not well understood (Sutherland et al., 2000) and may differ among populations.

Natal dispersal patterns differ between birds and mammals, where dispersal in birds is often female‐biased and male‐biased in mammals (Greenwood, 1980). In birds and mammals, three underlying ecological processes are thought to ultimately drive natal dispersal and observed dispersal differences between sexes: inbreeding avoidance, competition for resources, and competition for mates (Gaines & McClenaghan, 1980). Testing for proximate and ultimate drivers of natal dispersal within a theoretical framework can elucidate important ecological influences, how these may vary among populations, and identify potential conservation implications, particularly in threatened populations.

North American red squirrels (*Tamiasciurus hudsonicus*; hereafter red squirrels) are small (200–250 g) tree squirrels widespread throughout the coniferous forest regions of North America (Steele, 1998). Red squirrel ecology is conducive to investigating drivers of natal dispersal and settlement because red squirrels are diurnal, and both males and females defend a territory with a central larder hoard, or midden, making settlement obvious. Further, due to their widespread distribution, the ecology, life history, and natal dispersal have been documented in numerous red squirrel populations (Berteaux & Boutin, 2000; Haughland & Larsen, 2004a; Kemp & Keith, 1970; Kerr, Boutin, Lamontagne, McAdam, & Humphries, 2007; Larsen & Boutin, 1994; Steele, 1998; Sun, 1997).

In contrast to general mammalian dispersal patterns, natal dispersal in red squirrels is characterized as sex‐unbiased and tends to be philopatric (Larsen, 1993; Larsen & Boutin, 1998). Competition for resources best explained the observed patterns of sex‐unbiased red squirrel dispersal, and the continuous occupied habitat in most areas where red squirrels occur likely explains philopatric settlement (Larsen & Boutin, 1998). Territory acquisition and associated resources, including conifer cone storage (Williams, Lane, Humphries, McAdam, & Boutin, 2014), are critical to the survival and reproduction of both male and female red squirrels (Kemp & Keith, 1970; Larsen & Boutin, 1994, 1998; Rusch & Reeder, 1978), which may influence the tendency for both sexes to settle within or adjacent to their mother's territory (Berteaux & Boutin, 2000; Haughland & Larsen, 2004b; Kerr et al., 2007; Larsen & Boutin, 1998; Sun, 1997). While sex‐unbiased, philopatric dispersal appears common throughout the red squirrel's range, no data exist for isolated, peripheral tree squirrel populations. Peripheral, isolated populations could differ from range center populations due to environmental heterogeneity in availability of resources, landscape fragmentation, population dynamics, and local population density. Peripheral populations, in turn, may be influential in determining species distributions, and natal dispersal in these populations likely influences range expansion and contraction. Environmental heterogeneity may influence intrinsic characteristics of individuals within a population including body condition of mother and offspring (Bowler & Benton, 2005; Rémy, Le Galliard, Gundersen, Steen, & Andreassen, 2011) and individual personality (Cote et al., 2010), which can influence natal dispersal patterns.

**Figure 1:**
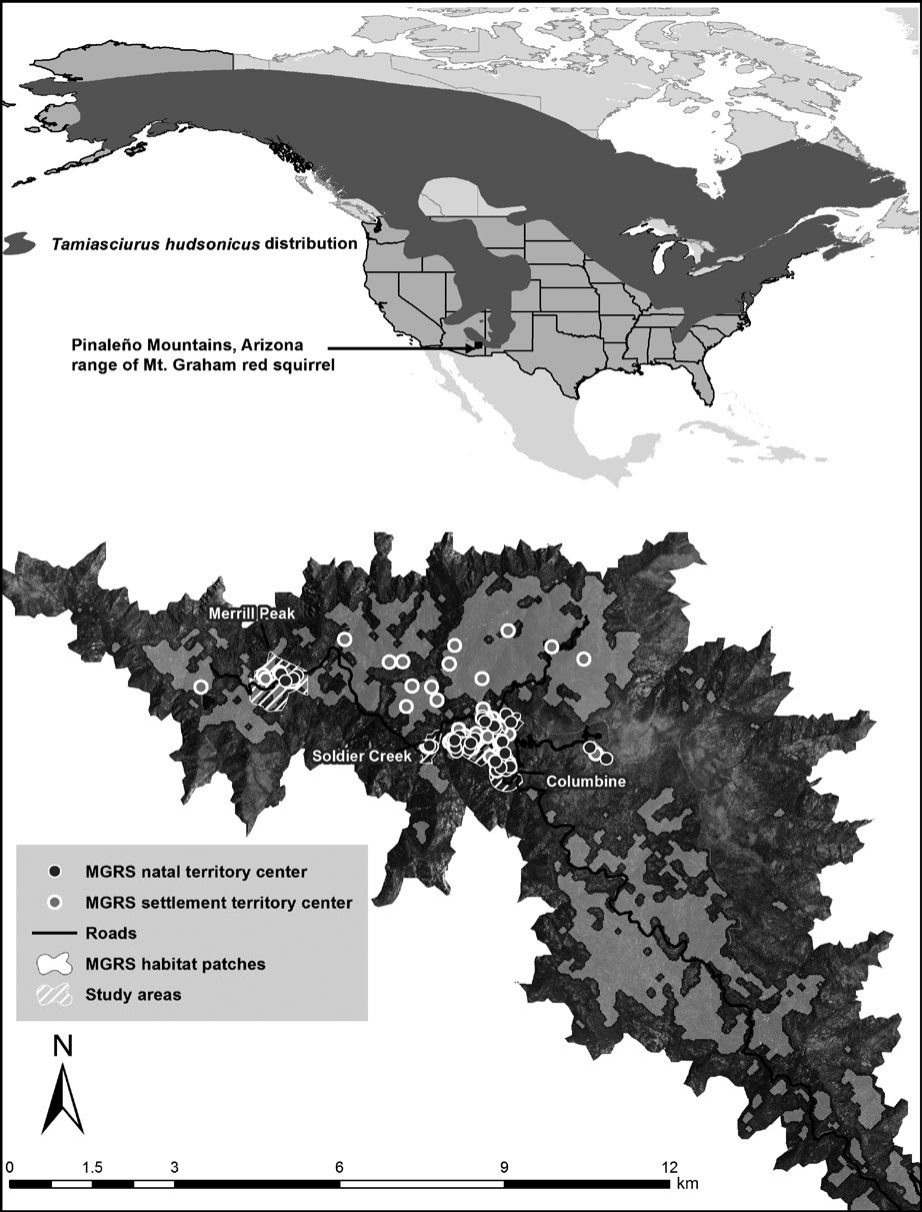
Distribution of North American red squirrels (*Tamiasciurus hudsonicus*) in North America (top inset) and Mt. Graham red squirrel (*T. h. grahamensis*) habitat in the Pinaleño Mountains, Arizona, USA. Study areas are shown in white hatching, natal territory centers, and settlement territory centers (2010–2013) indicated by gray and black circles, respectively, and outlines of habitat patches shown in gray polygons

Herein, we characterize natal dispersal in an isolated red squirrel subspecies occurring at the southern extent (trailing edge) of the species’ range (Figure 1) and compare dispersal in this isolated population to populations in the range center. We examine the influence of intrinsic and extrinsic factors on natal dispersal distance and the probability of nonphilopatric dispersal to include local population parameters (local male and female density), litter sex ratios, mother spring body mass, juvenile body condition, natal habitat patch size, and individual behavior traits. We develop a priori models to test support for three proximate factors and three ultimate ecological processes hypothesized to influence the probability of dispersing and dispersal distance: natal patch size, local density, individual behavior traits, inbreeding avoidance, competition for mates, and competition for resources (Greenwood, 1980; Larsen & Boutin, 1998).

### 1.1 Proximate hypotheses

Natal patch size: In highly fragmented landscapes, habitat patches might be smaller and farther apart. We examine the relationship between natal patch size and distance to the nearest patch and the probability of dispersing and dispersal distance. Local density: We test for positive and negative density dependence (e.g., Matthysen, 2005) based upon local density of occupied territories. Behavior: Personality traits may predispose some individuals to leave the natal area and disperse farther compared to others. We examine the relationship between individual behavior traits and the probability of dispersing and dispersal distance (Table 1).

**Table 1:** Matrix of variables measured to test support for ultimate hypotheses and proximate factors thought important for mammalian dispersal. The direction of the predicted relationship between a given variable and natal dispersal distance and probability of dispersal if a hypothesis is supported are indicated with + or −

	Variables examined							
	Resources	Density	Local demographics				Natal patch size	Behavior traits
Mammalian dispersal hypotheses	Juvenile body condition, mothe spring mass	Occupied middens/ha within 3.14 ha buffer	Middens occupied by males/ha	Middens occupied by females/ha	Proportion litter mates male	Proportion litter mates female	Patch area (ha), distance to patch, core or peripheral patch	Open field and mirror image stimulation behavior scores
Ultimate drivers								
Inbreeding avoidance								
M				+		+		
F			+		+			
Competition for resources								
M	+		+		+			
F	+			+		+		
Competition for mates								
M			+		+			
Proximate factors								
Natal patch size							−	
Local density								
Positive density dependence		+						
Negative density dependence		−						
Behavior traits								+/−

### 1.2 Ultimate hypotheses

Inbreeding avoidance: We examine the influence of local neighborhood and litter sex ratios on the probability of nonphilopatric dispersal and dispersal distance. Male competition for mates: We examine the influence of local male density and proportion of male littermates on the probability of nonphilopatric dispersal and dispersal distance. The promiscuous mating system in red squirrels and many other small mammals implies that there is likely little intrasexual competition for mates among females (Larsen & Boutin, 1998; Lawson Handley & Perrin, 2007). Competition for food: We examine the influence of resource proxies (spring body mass of the squirrel's mother and juvenile body condition) on the probability of nonphilopatric dispersal and dispersal distance (Table 1).

## 2 Materials and Methods

### 2.1 Study site and population

The Mt. Graham red squirrel (*Tamiasciurus hudsonicus grahamensis*, hereafter MGRS) is an endangered subspecies of red squirrel inhabiting the Pinaleño Mountains, in Arizona, USA, 32.7017°N, 109.8714°W, and is the southernmost population of red squirrels in North America (Sanderson & Koprowski, 2009; Figure 1). MGRS have been isolated for at least 10,000 years following post‐Pleistocene glacial retreat (Harris 1990) and are morphologically, vocally, and genetically distinct from the nearest subspecies of red squirrel, *T. h. mogollonensis*, inhabiting the White Mountains of east central Arizona (Fitak, Koprowski, & Culver, 2013; Koprowski, Alanen, & Lynch, 2005).

Our study areas comprise vegetation communities of mixed conifer forest dominated by Douglas fir (*Pseudotsuga menzesii*), southwestern white pine (*Pinus strobiformis reflexis*), white fir (*Abies concolor*), corkbark fir (*Abies lasiocarpa* var. *arizonica*), Engelmann spruce (*Picea engelmannii*), and aspen (*Populus tremuloides*) and spruce fir forest dominated by corkbark fir and Engelmann spruce (O'Connor, Falk, Lynch, & Swetnam, 2014; Smith & Mannan, 1994). Animals were captured primarily within three mixed conifer forest study sites: Columbine (104.3 ha; *n* = 83), Soldier Creek (14.7 ha; *n* = 6), and Merrill Peak (72.2 ha; *n* = 7; Figure 1). MGRS habitat in the Pinaleños occurs above 2,591 m, and animals in our study used habitat between 2,647 m and 3,267 m in elevation. Interannual availability of food resources from conifer seeds and fungi can vary by an order of magnitude, and total resource abundance has decreased following recent disturbance events (King & Koprowski, 2009; Koprowski et al., 2005). The Pinaleños have experienced patchy forest damage at varying levels of severity, due to insect infestations (Koprowski et al., 2005) and subsequent fires that burned a combined 14,160 ha of pine, mixed conifer, and spruce fir forest which, combined with tree death from insects, reduced MGRS habitat by 66% (O'Connor et al., 2014).

### 2.2 Live trapping and quantifying individual behavior traits

Between May 2010 and February 2014, we trapped, radio‐collared and tracked 94 juvenile (≤190 g) and four subadults (>190 g) MGRS through dispersal, settlement, and postsettlement. To capture juveniles, we monitored the location and reproductive condition of radio‐collared adult females as part of a long‐term study of MGRS space use (Koprowski, King, & Merrick, 2008). We observed lactating adult females at natal nests until juvenile emergence. Following emergence of juveniles, we set Tomahawk live traps (Tomahawk Live Trap, Tomahawk, WI, USA: model # 201) around the natal nest and midden between 0600 and 1800 hr to capture juveniles (≥90 g) with trap checks once per hour. Upon capture, we transferred each individual to a cloth handling cone to measure morphological traits, apply ear tags, and fit radio collars (Koprowski et al., 2008). To reduce radio collar weight and allow for growth, we used a thin (3 mm) nylon zip‐tie neck band with a 3 mm × 20 mm strip of thin, compressible foam mounting tape affixed to the inside of the neck band (total collar weight = 5 g; 3% of mean juvenile body mass, range: 2.5–5%). We recaptured individuals at least every 3 month to measure growth and check radio collar fit.

To characterize individual behavior traits that comprise personality, we performed two, 7.5‐min behavior trials on 84 juveniles at the site of capture: open field (OF) to quantify activity levels and exploration of a novel environment, and mirror image stimulation (MIS) to quantify aggression (Boon, Réale, & Boutin, 2007; Martin & Reale, 2008). We carried out behavior trials in a 40.6 cm × 54 cm × 54 cm collapsible arena constructed of white Makrolon^®^ extruded polycarbonate (Bayer MaterialScience LLC, Sheffield, MA, USA; designed and fabricated at Plastics, Inc., Tucson, AZ). The floor of the arena contains a removable panel with four blind holes for differentiating exploration and activity (Martin & Reale, 2008), and the rear wall of the arena has a sliding polycarbonate panel that can be removed to reveal a mirror, marking the transition between OF and MIS trials. The opaque lid of the arena contains a 5‐cm‐diameter hole through which we fit a USB web camera (Logitech QuickCam 960‐00‐247 Logitech, 7700 Gateway Blvd. Newark, CA 94560 USA, www.logitech.com).

We transferred marked individuals into the behavior arena and began recording the OF trial. After 7.5 min, we revealed the mirror, beginning the MIS trial. We recorded all digital videos with EvoCam software (Evological, www.evological.com) on a MacBook laptop (Apple, Cupertino, CA, USA). To reduce influence of outside noise and to standardize the arena experience for each animal (Svendsen & Armitage, 1973), we played an .MP3 audio track of a running stream (“Wilderness Creek,” www.naturesounds.ca) at full volume (60–65 db) for the entire behavior trial. Upon completion of the MIS trial, we released individuals and cleaned the entire arena with 90% isopropyl alcohol. We repeated behavior trials on a subset of 13 individuals between 6 weeks and 3 years after the original behavior trial to check the assumptions of repeatability of individual behavior traits. We tested most individuals once to reduce handling due to the federally endangered status of this population.

We scored digital video of OF and MIS behavior trials separately in JWatcher‐Video V1.0 software (Animal Behaviour Laboratory Macquarie University, Sydney Australia; Blumstein et al. 2006) and used ethograms similar to Boon et al. (2007) (Table S1). For each behavior trial, we summarized the proportion of time that an individual spent in each behavior state, or the number of times instantaneous events occurred (e.g., attacks on mirror).

### 2.3 Dispersal, density, and food

#### 2.3.1 Dispersal

We used digital receivers (Communication Specialists Inc. R‐1000 receiver) and yagi 3‐element directional antennae (Wildlife Materials Inc., Murphysboro IL, USA) to track juvenile MGRS movements from capture to settlement, locating each juvenile a minimum of 12 times monthly until settlement, death, or disappearance from our study area.

We monitored individuals for signs of settlement, which included conifer cone caching at a central midden (larderhoard) and territorial vocalizations (Larsen & Boutin, 1994). After settlement, we continued to monitor individual space use and survivorship. We measured straight‐line dispersal distance from the natal nest to the territory center (midden) at which it settled. In addition to dispersal distances quantified in this study (2010–2013), we also had 11 records of dispersal distances for animals ear‐tagged as juveniles in prior years (*n* = 8), and an early attempt to track natal dispersal in this population (*n* = 3; Kreighbaum & Van Pelt, 1996). We compiled published natal dispersal distances for red squirrels to identify range‐wide mean dispersal distance for males and females and compared range‐wide mean dispersal distances to dispersal distances in MGRS. Mean adult female 95% fixed kernel home range size in mixed conifer forest during fall (when juveniles settle) over 12 years was 1.7 ha: a territory diameter of 147.12 m, 73.56 m radius. We considered juveniles moving distances ≤150 m as settling within a territory contiguous with that of its mother (Larsen & Boutin, 1994), and juveniles moving distances >150 m as dispersers.

#### 2.3.2 Animal density

We determined occupancy of central larder hoards (middens) during quarterly censuses where we recorded signs of recent activity, including fresh conifer cone scales, digging, and cached cones and mushrooms (Koprowski & Snow, 2009) along with the age and sex of the resident. Between 2002 and 2015, mean adult female 95% fixed kernel home range size was 3.2 ha (range 1.1–7 ha), and we used this mean area to represent the local density that juveniles were exposed to prior to dispersal. We compiled occupancy of middens each quarter (December, March, June, and September) and determined local neighborhood density and sex ratios within a 100‐m‐radius (3.14 ha) buffers around natal nests by summarizing census occupancy records within each buffer. We used June census data to represent the density of occupied middens (where 1 occupied midden = 1 squirrel) and sex of residents within each 100‐m‐radius buffer, as summer is coincident with juvenile growth, development, and dispersal.

#### 2.3.3 Food availability

We quantified conifer cone availability in the natal area each fall via methods similar to Humphries and Boutin (2000) and Studd, Boutin, McAdam, Krebs, and Humphries (2014). We established linear transects 30 m long × 2 m wide in four cardinal directions centered upon an individual's natal nest. We then counted the number of cones on each live conifer >5 cm diameter at breast height visible from one vantage point. We summarized the mean number of cones per live tree within each plot, created an estimate of cones per hectare at each natal nest, and present a cone index = log (estimated cones/ha).

### 2.4 Natal patch size

To delineate patches of red squirrel habitat in the Pinaleños based on MGRS use, we developed a habitat suitability model based upon 9,424 MGRS juvenile lifetime telemetry locations relative to seven 25‐m‐resolution LiDAR‐derived raster layers that included percent canopy cover, mean tree height, standard deviation in tree height, total basal area, live basal area, slope, and elevation (Appendix S1). We followed Girvetz and Greco's (2007, 2009) patch morph algorithm in ArcGIS to create habitat patches with quality and marginal edge habitat delineated (Appendix S1). We used patch area in hectares, patch code (quality patch interior or edge) associated with each individual's natal and settlement location, and distance to the nearest patch as explanatory variables in subsequent natal dispersal models.

### 2.5 Statistical analyses

We used ArcGIS 10.1 (Environmental Systems Research Institute, Redlands CA) and R 3.3.1 (R Core Team 2016) for geospatial and statistical analyses. We based model comparisons on AIC_c_ values (Akaike information criterion adjusted for small samples size). We considered models with the lowest AIC_c_ score to be the top candidate models, and models with AIC_c_ score ≤2 from the top model were considered competing. Log‐transformed dispersal distance (m) better met the assumptions of normality so we used log‐transformed dispersal distance in subsequent models. Reported means are on raw data ± *SD* unless otherwise noted, statistical tests are based on α = 0.05, and distance units are in meters unless otherwise noted.

#### 2.5.1 Individual behavior

For both OF and MIS behavior trials, we collapsed the proportion of time spent in behaviors into synthetic variables, or principal components (PCs), via singular value decomposition of the centered, scaled data matrix (prcomp, R Core Team 2016). The first four principal components extracted from OF trials explained 75% of total variance; OFPC1 is positively weighted by inactivity, OFPC2 by climbing, OFPC3 by chewing and digging, and OFPC4 by locomotion. The first four principal components extracted from MIS trials explained 58% of total variance; MISPC1 is positively weighted by vigilance, while immobile, MISPC2 by mirror contact, MISPC3 by inactivity close to the mirror, and MISPC4 by climbing (Table S1). Based on univariate analyses, open field PC3 (OFPC3) “chew/dig” and PC4 (OFPC4) “locomotion” and mirror image stimulation PC2 (MISPC2) “alert mirror contact” and PC4 (MISPC4) “climb” had the most explanatory power with regard to dispersal distance (OFPC3 Pearson's *r* = .26; *p *=* *.06; OFPC4 Pearson's *r* = .26, *p = *.06; MISPC2 Pearson's *r* = .22, *p *=* *.13; MISPC4 Pearson's *r* = .14, *p = *.34); thus, we included these as intrinsic variables in subsequent models.

We estimated the repeatability of individual behavior scores by selecting a subset of individuals (*n* = 13) to receive repeat OF and MIS trials. We compiled behavior data from trials 1 and 2 for each individual, and collapsed variables via principal component analysis as above. We calculated the intraclass correlation coefficient (ICC) and confidence intervals (α = 0.05) for principal components from OF and MIS trials, with animal ID as the subject, and *n* = 2 “raters” (trial 1 and trial 2), and specified a test for consistency between trials (model one, one way), where subject effects are random (Gamer, Lemon, Fellows, & Singh, 2015).

#### 2.5.2 Models for dispersal distance and probability of long‐distance dispersal

We examined the influence of environmental and intrinsic factors on natal dispersal distance and probability of nonphilopatric dispersal within three model sets: sexes combined, female‐only, and male‐only models (Tables 3 and S2). Our candidate model sets contained five basic models: (1) null (intercept only), (2) global (all variables included, *k* = 13), (3) extrinsic (mother spring mass, proportion of male/female littermates, occupied middens per hectare, middens occupied by males per hectare, middens occupied by females per hectare, log of natal patch area, patch code), (4) intrinsic (body condition index, OFPC3 and 4, MISPC 2 and 4), and (5) natal patch fragmentation (log patch area, patch code) models (see Table S3 for descriptions). Female‐ and male‐specific models included the same five basic models in addition to models developed to specifically test dispersal hypotheses (density, natal patch size, behavior, inbreeding avoidance, competition for mates, competition for resources; Tables 3 and S2) in either sex. We identified 16 candidate generalized linear models a priori to test for the influence of intrinsic and extrinsic factors on log‐transformed straight‐line dispersal distance (Gaussian error structure), and on the probability of dispersing long distances (logit link, binomial error structure; Table S2), and compared models, fit with maximum‐likelihood, within an information‐theoretic model selection framework. For probability models, we specified nonphilopatric dispersal for males ≥150 m (>total diameter of mean adult female home range) and ≥100 m for females (as few females dispersed >150 m).

## 3 Results

### 3.1 Live trapping and individual behavior differences

Of the 98 radio‐collared juvenile and subadult MGRS in our study (51 females and 47 males), 12 died prior to settlement (nine females and three males) and 24 had unknown fates (14 females and 10 males); of these, seven collars were found (four females and three males) and 17 went missing and were never relocated (nine females and seven males). Sixty‐three individuals survived and were successfully tracked to settlement locations (29 females and 34 males). Combined with known dispersal distances from previous years (six males and five females), we were able to quantify dispersal distance for 74 MGRS.

We quantified individual behavior traits via open field and mirror image stimulation in 85 of 98 radio‐collared juvenile and subadult MGRS, with a limited subset of 13 repeat behavior trials (*n* = 2 trials/individual); time between trials ranged from 38 to 1,092 days. Repeatability of behaviors in MGRS can be classified as slight to fair with intraclass correlation coefficient values ranging from 0 to 0.3, where <0.00 = poor agreement, 0.0–0.20 = slight agreement, 0.21–0.40 = fair, 0.41–0.60 = moderate, 0.61–0.80 = substantial, 0.81–1.00 = almost perfect (Landis & Koch, 1977); (OFPC1: ICC = −0.68, *F*_12,13_ = 0.18, *p *=* *.99; OFPC2: ICC = 0.05, *F*_12,13_ = 1.11, *p *=* *.42; OFPC3: ICC = 0.15, *F*_12,13_ = 1.36, *p *=* *.29; OFPC4: ICC = 0.12, *F*_12,13_ = 1.27, *p *=* *.34; MISPC1: ICC = 0.32, *F*_12,13_ = 1.92, *p *=* *.13; MISPC2: ICC = 0.01, *F*_12,13_ = 1.11, *p *=* *.48; MISPC3: ICC = −0.08, *F*_12,13_ = 0.84, *p *=* *.61; MISPC4: ICC = 0.20, *F*_12,13_ = 1.48, *p *=* *.25).

### 3.2 Dispersal, density, food, and natal patch size

#### 3.2.1 Dispersal

**Figure 2:**
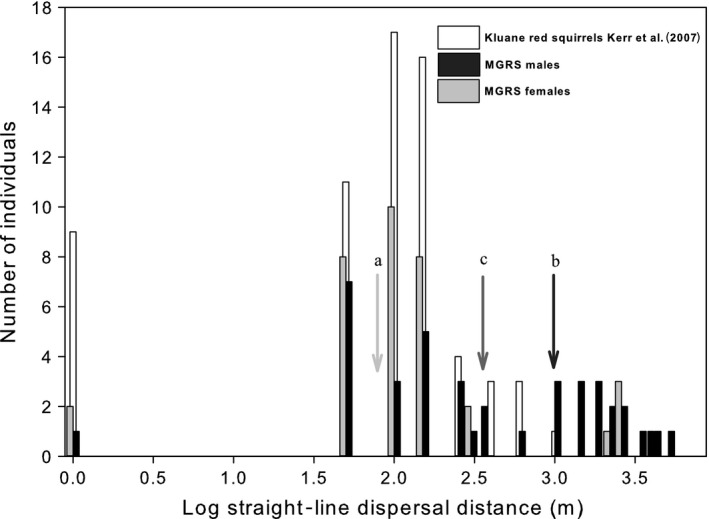
Frequency distribution of straight‐line dispersal distances (1996–2013) for juvenile Mt. Graham red squirrel (*T. h. grahamensis*) males (black) and females (gray), left axis, compared to frequency distribution of juvenile North American red squirrel (*Tamiasciurus hudsonicus*) straight‐line log dispersal distances reported for 67 individuals from the Yukon (Kerr et al., 2007), right axis. Mean dispersal distance for Yukon red squirrels, MGRS males, and females is indicated by arrows a, b, and c, respectively. MGRS male mean dispersal distance = 969.4 m ± 1224.8; MGRS females = 339.0 m ± 726.4, Yukon males and females = 92.4 m ± 123.3

Natal dispersal in MGRS is male‐biased with exaggerated dispersal distances compared to other red squirrel populations (Table 2) and greater than reported for 64 juvenile red squirrels from the Yukon (mean dispersal distance: Yukon red squirrels = 92.4 m ± 123.3; MGRS: 679.8 ± 1067.7; Welch *t*_97.6_ = −2.5, *p *=* *.02; Kerr et al., 2007; Figure 2). Male MGRS dispersed farther than females (mean dispersal distance: males = 969.4 m ± 1224.8; females = 339.0 m ± 726.4; Welch *t*_64.8_ = −2.4, *p *=* *.02). Across years, 53% of juveniles exhibited dispersal: 41% of females ≥100 m, 18% of females ≥150 m, and 65% of males ≥150 m annually.

**Table 2:** Comparison of natal dispersal distances reported for North American red squirrels (*Tamiasciurus hudsonicus*) throughout their range. Means ± standard deviations are provided where available

Mean dispersal distance (m)	Range (m)	Males	Females	*N*	Habitat type; region	Source	Notes
~1,600	NA	NA	NA	8	aspen (*Populus tremuloides*)–spruce (*Picea* spp); Alberta	Kemp & Keith (1970)	In 1967, eight of nine marked juveniles dispersed “about 1.6 km,” and in 1968 three marked juvenlies remained philopatric.
273.3^a^	NA	247.7 ± 43.3^a^	298.8 ± 61.7^a^	55	jack pine (*Pinus banksiana*)–spruce (*Picea* spp); Alberta	Larsen (1993)	Distance is mean maximum distance moved (including forays), with no observed difference between sexes. Mann–Whitney *U*‐test, *Z* = −0.46, *p *=* *.64
88.6	0.0–323.0	85.1	86.9	73	jack pine (*Pinus banksiana*)–spruce (*Picea* spp); Alberta	Larsen & Boutin (1994); Larsen (1993)	Mean settlement distance, no observed difference between sexes. Mann–Whitney *U*‐test, *Z* = −0.74, *p *=* *.46
178.8	NA	115.0	242.5	8	spruce (*Picea* spp)–fir (*Abies* spp); Minnesota	Sun (1997)	
NA	NA	NA	NA	73	jack pine (Pinus banksiana)–spruce (Picea spp); British Columbia	Larsen & Boutin (1998)	Distances not given specifically, but statistical tests show no sex bias, supporting resource competition hypothesis
96 ± 94	0.0–600.0	107 ± 111	85.0 ± 72.0	189	white spruce (*Picea glauc*a); Yukon	Berteaux & Boutin (2000)	Dispersal distance is only for successful dispersers, not philopatric individuals
86.0	0.0–4500.0	NA	NA	37	Douglas fir (*Pseudotsuga menziesii*); British Columbia	Haughland and Larsen, (2004b)	70 ± 10 mature forest, 79 ± 54 mature edge, 86 ± 46 thinned forest, 109 ± 31 thinned edge
92.4 ± 123.3	1.3–794.3	NA	NA	65	White spruce (*Picea glauc*a); Yukon	Kerr et al. (2007)	17 juveniles from food—supplemented mothers, 50 juveniles from control mothers
679.8 ± 1067.7	0.0–4788.0	969.4 ± 1224.8	339.0 ± 726.4	73	Mixed conifer; Arizona	Present study	73 juvenile Mt. Graham red squirrels
Mean dispersal distance for range center red squirrel populations excluding Kemp & Keith, 1970;						108.4 m	
Mean dispersal distance for range center red squirrel populations including Kemp & Keith, 1970						357.0 m	

a Indicates maximum distance moved (including forays), not included in calculation.

**Figure 3:**
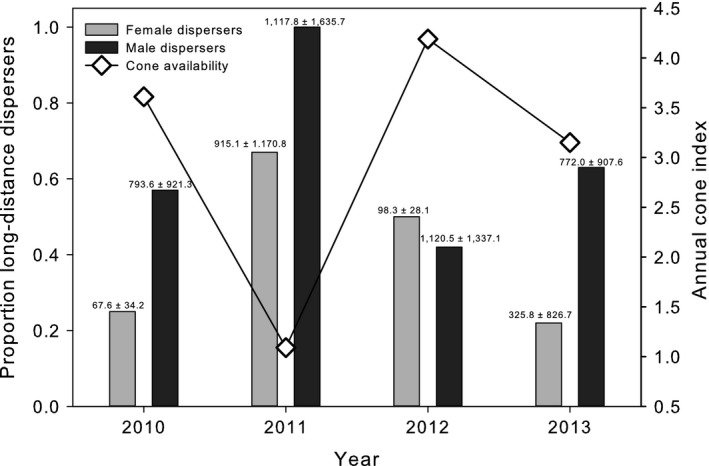
Proportion of male and female juvenile Mt. Graham red squirrels (*T. h. grahamensis*) making long‐distance dispersal movements (males dispersing ≤150 m; females dispersing ≤100 m) relative to an annual index of the current year's conifer cone availability (2010–2013). Proportion of female long‐distance dispersers for each year is indicated with gray bars, males with black bars. Mean ± standard deviation in dispersal distance for males and females for each year is shown above the bars

The proportion of juveniles that are nonphilopatric and distances moved varied from year to year (proportion males $\chi_{3df}^{2}$_*df*_ = 6.45, *p *=* *.09; proportion females $\chi_{3df}^{2}$ = 3.99, *p *=* *.26; all individuals $\chi_{3df}^{2}$ = 7.56, *p *=* *.06), and this interannual variation in dispersal may be influenced by conifer seed crop availability (Table S4, Figure 3). For both sexes, the proportion of individuals dispersing and female dispersal distance was highest in 2011 (proportion males: 1.0, proportion females: 0.67, female mean dispersal distance: 915.1 m), a year of lowest food availability (Table S4, Figure 3).

#### 3.2.2 Density

Density of MGRS in the Pinaleños is lower than reported in other red squirrel populations, in both spruce fir and mixed conifer forest types. Overall mean density on our long‐term study areas between fall 1989 and winter 2013 was 0.35 ± 0.2 and 0.18 ± 0.2 squirrels/ha in mixed conifer and spruce fir forest, respectively, compared to 1.34 ± 0.6, 2.9 ± 1.2, and 2.35 ± 0.02 squirrels/ha in mixed conifer and spruce forest reported for areas within core red squirrel range (Dantzer, Boutin, Humphries, & McAdam, 2012; Rusch & Reeder, 1978; Wheatley, Larsen, & Boutin, 2002) (mixed conifer: Welch's *t*_4.1_ = −3.62, *p *=* *.022; spruce fir: Welch's *t*_10.1_ = −5.9, *p *<* *.001).

During our study, local midden density within a 3.14‐ha buffer surrounding natal nests ranged from 0.3 to 3.8 middens/ha (mean 2.1 ± 0.9), and local occupancy ranged from 0.0 to 1.9 occupied middens/ha (mean 0.8 ± 0.4) and this value did not vary significantly by year (one‐way ANOVA *F*_1,58_ = 0.28, *p *=* *.60). The mean proportion of occupied middens within 3.14‐ha buffers was 0.37 ± 0.19.

#### 3.2.3 Current year's food

Cone availability varied among years with considerable variation among sites each year [year: mean visible cones/viable tree ± *SD*, range (2010: 14.4 ± 14.1, 1.2–38.5; 2011: 0.6 ± 1.0, 0.0–2.3; 2012: 37.5 ± 24.8, 7.8–82.5; 2013: 6.1 ± 6.7, 0.3–17.8)]. Our estimated cone index (log cones/ha) differed among years (Kruskal–Wallis rank sum test: $\chi_{3df}^{2}$ = 36.8, *p *<* *.001), with almost no cone production in 2011 (Figure 3).

#### 3.2.4 Natal patch size

Animals in our study were born in habitat patches ranging in size from 1.88 to 126.60 ha (mean 26.72 ± 26.00). The majority of individuals that dispersed and settled (66%) were born in small patches <30 ha in size, and 88% were born in patches designated as “quality patch interior” (90% of cells within a 50 m search radius of any focal cell are classified as suitable). Natal patches within our study areas were all <50 m of the next nearest patch; thus, we omitted distance to nearest patch from our models.

### 3.3 Drivers and tests of mammalian dispersal hypotheses

**Figure 4:**
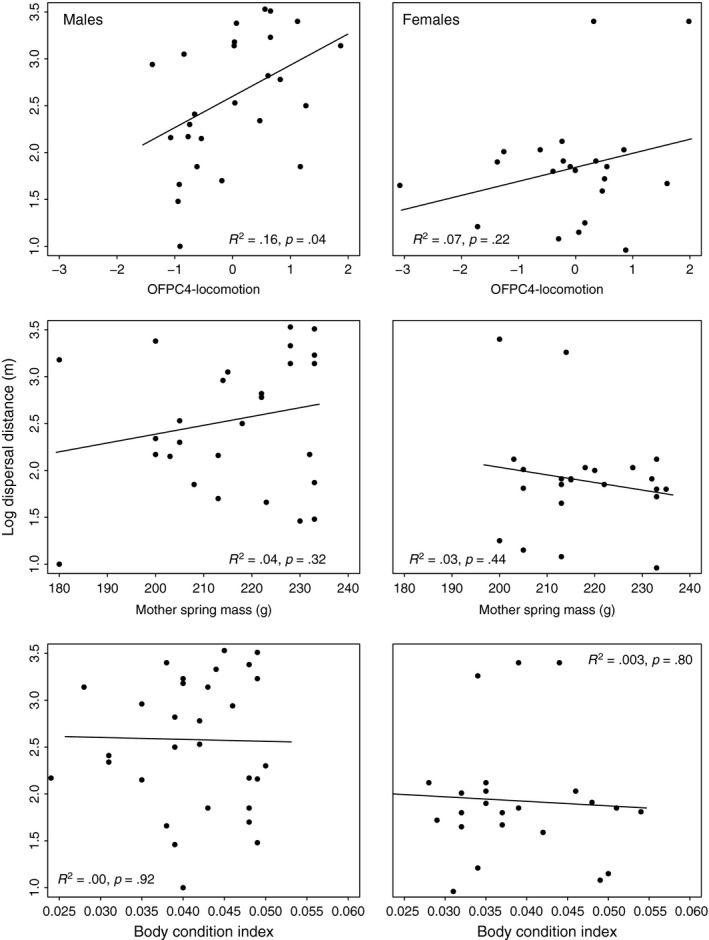
Linear relationships between variables included in our top model (resources.locomotion: mother spring mass, body condition index, and individual activity score, Tables 3 and 4) and juvenile Mt. Graham red squirrel (*T. h. grahamensis*) dispersal distance (2010–2013). MGRS males are represented in the left‐hand panel and females in the right‐hand panel

Across sexes, the most supported model explaining dispersal distance and probability of nonphilopatric dispersal was the saturated, global model (AIC weight = 0.99), indicating a combination of intrinsic and extrinsic factors influence natal dispersal distances. When we considered males and females separately, the top model explaining dispersal distance and probability of nonphilopatric dispersal in both sexes included mother spring mass, juvenile body condition, and activity (female AIC weight = 0.84, male AIC weight = 0.83; Tables 3 and 4). In dispersal distance models, the relationship between dispersal distance, mother spring mass, and individual body condition is reversed for males and females, with female dispersal distance negatively influenced by increases in mother spring mass and individual body condition, whereas male dispersal distance is positively influenced by increases in both activity and mother spring mass (Figure 4, Table 4). For females, the top probability of dispersal model (resources.locomotion; AIC weight 0.68; Table 3) was similar to the dispersal distance (resources.locomotion) model in both significance and sign of coefficients, but this was not the case for males (Table 4). For males, the coefficient for body condition is reversed in the top probability of dispersal model (resources.locomotion; AIC weight 0.74), but both body condition and mother spring mass had very little explanatory power, as this model is driven primarily by activity, whereby for every unit increase in movement PC score, males are over seven times more likely to disperse long distances (Table 4, Figure 4).

**Table 3:** Model descriptions and multimodel selection results for models developed a priori to explain dispersal distance and probability of long‐distance dispersal in juvenile Mt. Graham red squirrels (*Tamiasciurus hudsonicus grahamensis*) between 2010 and 2013. Models with AICc weights >0 are shown. Models developed to test for dispersal hypotheses are indicated: DEN = local density, FRAG = habitat fragmentation, BEHAV = individual behavior differences, CFR = competition for resources, IA = inbreeding avoidance. See Table S3 in electronic supplementary materials for all models

Model name	Dispersal hypothesis	K	AICc	Delta AICc	AICc Wt.	Cum.Wt.	LL	Evidence ratio
	**Response = log dispersal distance, Gaussian error structure**							
General models both sexes								
Global		13	89.43	0	0.99	0.99	−24.13	
Intrinsic		7	99.96	10.53	0.01	1	−41.50	193.25
Female models								
female.resources.locomotion	CFR, BEHAV	5	35.82	0	0.82	0.82	−9.91	
female.resources	CFR	4	39.98	4.16	0.1	0.93	−14.56	8.01
female.resources*density	CFR, DEN	5	43.41	7.59	0.02	0.95	−14.94	
female.resources.territories	CFR	5	43.73	7.91	0.02	0.96	−14.56	
female.resources.behavior	CFR, BEHAV	8	43.97	8.15	0.01	0.98	−3.70	
female.mother.mass	CFR	3	44.24	8.43	0.01	0.99	−18.49	
female.locomotion	BEHAV	3	45.21	9.39	0.01	0.99	−18.94	
Male models								
male.resources.locomotion	CFR, BEHAV	5	56.22	0	0.82	0.82	−21.23	
male.locomotion	BEHAV	3	59.66	3.44	0.15	0.97	−26.31	5.58
male.resources.behavior	CFR, BEHAV	8	65.34	9.12	0.01	0.98	−19.13	
male.resources	CFR	4	65.48	9.27	0.01	0.99	−27.79	
	**Response = binary long‐distance dispersal (≥150 m males, ≥100 m females), binomial error structure**							
General models both sexes								
Global		11	52.77	0	0.98	0.98	−10.31	
Intrinsic		6	61.05	8.27	0.02	1	−23.45	62.61
Female models								
female.resources.locomotion	CFR, BEHAV	4	25.48	0	0.62	0.62	−6.92	
female.resources	CFR	3	27.72	2.25	0.2	0.82	−10.06	3.07
female.locomotion	BEHAV	2	29.97	4.5	0.06	0.88	−12.67	
female.resources.territories	CFR	4	30.98	5.5	0.04	0.92	−10.06	
female.resource.competition	CFR	3	32.4	6.92	0.02	0.94	−12.60	
Extrinsic		7	33.04	7.56	0.01	0.95	−5.21	
female.bci	CFR	2	33.21	7.74	0.01	0.97	−14.31	
female.mother.mass	CFR	2	33.81	8.34	0.01	0.98	−14.61	
female.resources*density	CFR	4	34.13	8.66	0.01	0.98	−11.96	
female.density	CFR, DEN	2	34.21	8.74	0.01	0.99	−14.85	
Male models								
male.resources.locomotion	CFR, BEHAV	4	31.39	0	0.74	0.74	−10.52	
male.locomotion	BEHAV	2	33.6	2.21	0.24	0.98	−14.55	3.02

Model name	Variables
Global	mother.spring.mass, ppn.female, occ.mids.ha, occ.male.ha, occ.female.ha, bci, MIS2, MIS4, OF3, OF4, logpatch.area
Intrinsic	bci, MIS2, MIS4, OF3, OF4
Extrinsic	mother.spring.mass, ppn.female, midocc.ha, occmale.ha, occfemale.ha, logpatch.area
Null	intercept only
bci	bci
female.density	occ.female.ha
female.inbreeding	ppn.male, occ.male.ha
female.resource.competition	ppn.female, occ.female.ha
female.resources*density	mother.spring.mass, occ.female.ha, mother.spring.mass*occ.female.ha
Locomotion	OF4
male.inbreeding	ppn.female, occ.females.ha
mother.mass	mother.spring.mass
natalpatch	logpatch.area, patch.code
resources	mother.spring.mass, bci
resources.behavior	mother.spring.mass, bci, MIS2, MIS4, OF3, OF4
resources.locomotion	mother.spring.mass, bci, OF4
resources.territories	mother.spring.mass, occ.mids.ha, bci

**Table 4:** Model coefficients for the top model (resources.locomotion) explaining dispersal distance and probability of long‐distance dispersal in juvenile male and female Mt. Graham red squirrels (*Tamiasciurus hudsonicus grahamensis*) between 2010 and 2013

Model variables	Male model coefficients					Female model coefficients				
	β	± *SE*	95% C.I.	*p*	odds ratio	β	± *SE*	95% C.I.	*p*	Odds ratio
Dispersal distance										
bci	3.08	20.77	−37.63 to 43.79	.88		−29.58	20.19	−69.15 to 9.99	.17	
mother.spring.mass	0.01	0.01	−0.01 to 0.03	.39		−0.03	0.01	−0.06 to 0.00	.06	
OF4	0.36	0.19	−0.01 to 0.74	.07		0.18	0.16	−0.13 to 0.50	.27	
Long distance										
bci	−2.38	76.08	−159.66 to 157.37	.98	0.09	−201.64	115.15	−505.44 to −7.43	.08	0.00
mother.spring.mass	0.00	0.03	−0.07 to 0.06	.92	1.00	−0.16	0.09	−0.43 to −0.02	.09	0.85
OF4	1.98	0.88	0.51 to 4.10	.02	7.22	1.23	1.02	−0.33 to 4.19	.22	3.44

**Figure 5:**
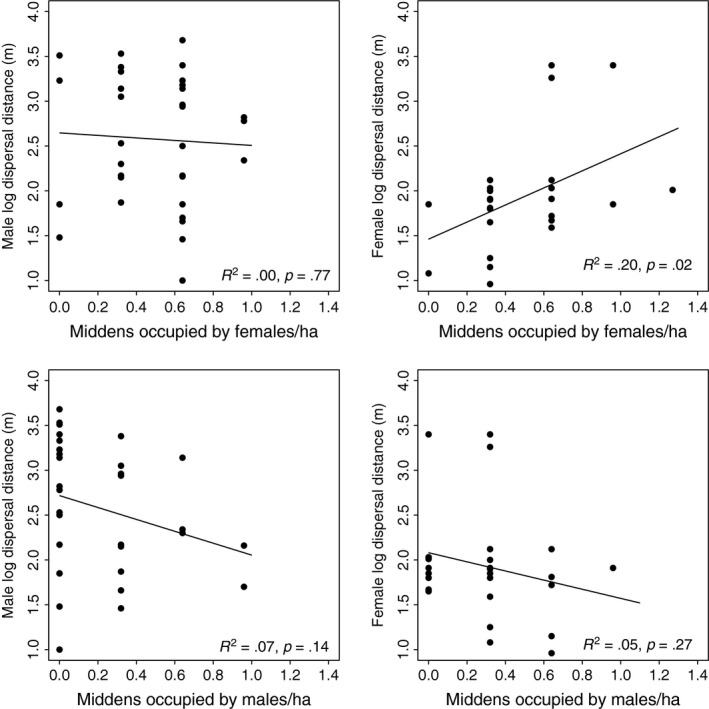
Linear relationships between inter‐ and intrasexual local population density (local density of middens occupied by males and females) and juvenile Mt. Graham red squirrel (*T. h. grahamensis*) dispersal distance (2010–2013). These relationships between dispersal distance and local conspecific and heterospecific density are of interest despite having less support within a multimodel selection framework as they demonstrate lack of support for the inbreeding avoidance hypothesis and suggest possible competition for resources among females

We found no support for local density and natal patch size as proximate explanatory factors or inbreeding avoidance as an ultimate driver of dispersal distance or probability of dispersal (Table S2). Evidence for an intrasexual effect of female density on female dispersal distance exists, whereby juvenile females dispersed farther with increasing local female density (Figure 5), yet despite this relationship, female density was not a top model (Tables 3 and S2). Male dispersal distance was not influenced by local female density, further evidence against current inbreeding avoidance (Figure 5). We found no relationship between mother spring mass and litter sex ratio (proportion male offspring) (*t *=* *0.06, *df* = 47, *p = *.95) or an effect of year (*F*_3,54_ = 0.99, *p *=* *.40).

## 4 Discussion

Natal dispersal in MGRS is sex‐biased, and juveniles disperse up to nine times farther than other North American red squirrel populations. This system provided a unique opportunity to examine potential intrinsic and extrinsic factors and evolutionary drivers associated with dispersal in this unique population. The Pinaleño Mountains, at 32°N latitude, represent a biogeographically distinct landscape compared to 53°N latitude, in Alberta, Canada, near the centroid of red squirrel range in North America. Population density, survival, and life expectancy (<2 years; Goldstein, Merrick & Koprowski 2016) are much lower, and home ranges nearly 10 times larger in the Pinaleños (Koprowski et al., 2008), suggesting altered population dynamics, life history, or distribution of resources may play a role in natal dispersal differences observed in peripheral populations. Here, we provide evidence that natal dispersal in MGRS is most influenced by individual behavioral tendencies for exploratory movement and resources reflected by maternal spring body mass, individual juvenile body condition, and conifer seed crop abundance; competition for food resources is the most supported ultimate hypothesis. We found little support for proximate influences of natal patch size, local density, litter sex ratios, or ultimate drivers competition for mates and inbreeding avoidance.

### 4.1 The role of behavior and resources on dispersal distance

Individual behavior differences, or personalities, and their associated behavioral syndromes (correlated behavior traits) have been documented in many taxa (Sih, Cote, Evans, Fogarty, & Pruitt, 2012), including red squirrels (Boon, Réale, & Boutin, 2008; Boon et al., 2007; Kelley, Humphries, McAdam, & Boutin, 2015), and are thought to be maintained within populations by differential fitness relative to highly variable resource availability and population densities in time and space (Cote et al., 2010; Duckworth, 2008; Wolf & Weissing, 2012). Positive correlations between behavior traits and dispersal distance have been documented in birds, mammals, lizards, and fishes (Clobert et al., 2009; Cote et al., 2010; Dingemanse et al., 2003; Duckworth, 2008) and may be important in population dynamics and maintenance of gene flow, especially for species threatened with habitat shifts or other disturbances (Massot, Clobert, & Ferrière, 2008; Sih et al., 2012). Natal dispersal distance in MGRS is correlated with an individual's tendency to actively explore a novel environment. Vagile behavior trait expression appears to be mediated by external cues from mothers and the surrounding environment related to resource availability, an example of condition dependence and phenotype dependence (Clobert et al., 2009; Cote et al., 2010).

The competition for resources hypothesis implies that sex‐biased dispersal should occur only if resources are more important to one sex (the philopatric sex) than the other (Greenwood, 1980; Larsen & Boutin, 1998). For most mammals, including Sciurids (but *not* observed in red squirrels), this dichotomy between the resource needs of females (competition for resources) and males’ need for access to mates (competition for mates) has explained the primarily male‐biased dispersal patterns observed in mammals (Clutton‐Brock & Harvey, 1978; Greenwood, 1980). In red squirrels, acquiring a quality territory that supports the accumulation of food resources is critical for overwinter survival in both males and females (Kemp & Keith, 1970; Larsen & Boutin, 1994, 1998; Rusch & Reeder, 1978), and as breeding does not occur until after a juvenile's first winter (Koprowski, 2005), it follows that natal dispersal and settlement decisions are driven by resource availability (competition for resources) rather than mates (Larsen & Boutin, 1998). Our models, along with evidence of increased dispersal in years of low conifer cone abundance, support the finding that resource availability is an important driver of natal dispersal in MGRS. However, resources available to mothers prior to parturition, partially reflected in mother spring mass and subsequent juvenile body condition, appear to influence dispersal in males and females differently, contributing to the nonphilopatric, sex‐biased dispersal observed in MGRS.

### 4.2 Maternal influences on natal dispersal in a highly variable world

Maternal influence on offspring phenotype is widespread in mammals (Maestripieri & Mateo, 2009), and maternal effects are shown to influence offspring behavior and propensity for dispersal in response to resource variability (Duckworth, 2009). In North American red squirrels, a female's body mass following winter is a reflection of her territory quality and the number of conifer cones she was able to collect from her territory and hoard the previous fall (Becker, Boutin, & Larsen, 1998). External influences such as resource availability and competition for resources affect maternal condition and subsequent behavioral and physiological phenotypes and sex ratios in a female's offspring (Love & Williams, 2008; Maestripieri & Mateo, 2009). In this study, we observed no offspring sex ratio differences and no relationship between mother's mass or year and offspring sex ratio, and provide evidence for maternal effects that may maximize the fitness of both sons and daughters within a highly variable environment. The positive associations between dispersal distance and mother spring body mass and dispersal distance and individual activity score in males provide some evidence that mothers in good condition tend to have active, exploratory sons that were long‐distance dispersers. The negative relationship between dispersal distance and mother spring body mass in females provides some evidence that the same mothers in good condition tend to have philopatric daughters. In poor years, the majority of all offspring dispersed. In years of high‐resource abundance, allowing daughters to settle adjacent to or within a quality territory increases overall fitness of both mother and daughter (Berteaux & Boutin, 2000). Such resource‐ and density‐mediated adjustments in offspring sex ratios via natal dispersal could represent a flexible (rather than fixed) dispersal strategy that is adaptive in a highly variable environment.

### 4.3 Insight from the range periphery

Ecological conditions such as extreme heterogeneity in resource availability characteristic of trailing edge peripheral populations can contribute to heterogeneous dispersal patterns among populations. Intraspecific variation in dispersal distance has been documented in arvicoline rodents and suggests that while most studies report short dispersal distances, long‐distance dispersal events, while infrequent, can occur (Le Galliard et al., 2012).

Peripheral populations represent microcosms of evolution, with distinct physical, physiological, and behavioral adaptations resulting from long‐term isolation and environmental conditions different from the range center (Channell & Lomolino, 2000; Foster, 1999; Hampe & Petit, 2005). Compared to range center, peripheral populations exhibit decreased densities (Lomolino & Channell, 1995), expanded home range size (Koprowski et al., 2008), lower within‐population genetic diversity (Fitak et al., 2013; Vucetich & Waite, 2003), variation in demographic parameters, and changes in the frequency of behaviors or shifts in behavior reaction norms (Foster, 1999). Understanding how environmental variables and individual phenotypes influence natal dispersal across a species’ range is therefore of special interest for predicting how populations may respond to environmental change (Channell & Lomolino, 2000; Hampe & Petit, 2005; Woolbright, Whitham, Gehring, Allan, & Bailey, 2014).

## Data Accessibility

Data are archived in Dryad (http://datadryad.org/), doi:10.5061/dryad.ts51k)

## Conflict of Interest

None declared.
