# Supplementary material for: Altered natal dispersal at the range periphery: The role of behavior, resources, and maternal condition
Source: Ecol Evol. 2016 Nov 30;7(1):58–72. doi: 10.1002/ece3.2612 (PMC5216619; doi:10.1002/ece3.2612)
Supplement: Supplementary file 1 [file ECE3-7-58-s001.docx]

Table S1. Ethogram for open field (OF) and mirror-image stimulation (MIS) behaviour trials on juvenile Mt. Graham red squirrel (*Tamiasciurus hudsonicus grahamensis*) between 2010 and 2013. Associated loadings for principal components most associated with dispersal movement from OF and MIS trials (OF 3 & 4; MIS 2 & 4) are shown with the largest, positive loadings indicated in bold.

|  | **Open Field Trial** | | | | |
| --- | --- | --- | --- | --- | --- |
| **Behaviour** | **Behavior description** | **OF1 Stillness** | **OF2 Climbing** | **OF3 Chew/Dig** | **OF4 Locomotion** |
| Chew/dig | Chew or scratch floors or walls | -0.46 | -0.02 | **0.36** | -0.48 |
| Climb/hang | Climbing or hanging on walls | -0.05 | **0.45** | -0.66 | 0.09 |
| Grooming | Grooming activity | 0.15 | -0.48 | -0.01 | 0.22 |
| Head dip | Dip head into blind holes | -0.15 | -0.38 | -0.10 | -0.39 |
| Immobile | No movement | **0.55** | 0.01 | 0.15 | -0.31 |
| Locomotion | Jump, walk | -0.43 | -0.09 | 0.28 | **0.61** |
| Rear | Rising up on hind legs | -0.21 | -0.28 | -0.43 | -0.20 |
| Scan | Head moving, rest of body immobile | **0.44** | -0.30 | 0.06 | 0.22 |
| Sniff | Sniff floor or walls | -0.12 | -0.49 | -0.36 | 0.06 |
|  | Proportion of variance explained | 0.28 | 0.25 | 0.11 | 0.11 |
|  | Cumulative proportion | 0.28 | 0.53 | 0.64 | 0.75 |
|  |  |  |  |  |  |
|  | **Mirror Image Stimulation Trial** | | | | |
| **Behaviour** | **Behavior description** | **MIS1 Sill/Alert** | **MIS2 Mirror Contact** | **MIS3 Still/Front** | **MIS4 Climbing** |
| Chew/dig | Chew or scratch floors or walls | -0.48 | -0.09 | -0.01 | 0.02 |
| Climb/hang | Climbing or hanging on walls | 0.05 | -0.13 | -0.09 | **0.76** |
| Crouch | Attack imminent - tail over back with hairs erect | 0.06 | **0.34** | -0.22 | 0.12 |
| Grooming | Grooming activity | -0.19 | 0.28 | 0.01 | -0.36 |
| Locomotion | Jump, walk | -0.45 | 0.06 | -0.06 | -0.02 |
| Non-aggressive | non aggressive contact with mirror | -0.15 | **0.38** | 0.03 | 0.19 |
| Number attacks | Count of attacks on mirror | -0.10 | **0.36** | -0.15 | 0.12 |
| Rear | Rising up on hind legs | -0.36 | 0.21 | 0.10 | 0.01 |
| Scan | Head moving, rest of body is immobile | **0.43** | **0.35** | -0.08 | -0.13 |
| Slow approach/stretch | Slow approach towards mirror, back legs stretched out behind | **0.24** | **0.33** | -0.21 | 0.26 |
| Sniff | Sniff floor or walls | -0.01 | **0.45** | 0.17 | -0.14 |
| Still in back | Immobile in back half of arena furthest from mirror | 0.15 | -0.19 | -0.63 | -0.35 |
| Still in front | Immobile in front half of arena closest to mirror | **0.29** | -0.01 | **0.65** | -0.07 |
|  | Proportion of variance explained | 0.22 | 0.13 | 0.12 | 0.1 |
|  | Cumulative proportion | 0.22 | 0.36 | 0.48 | 0.58 |
