# Supplementary material for: Altered natal dispersal at the range periphery: The role of behavior, resources, and maternal condition
Source: Ecol Evol. 2016 Nov 30;7(1):58–72. doi: 10.1002/ece3.2612 (PMC5216619; doi:10.1002/ece3.2612)
Supplement: Supplementary file 2 [file ECE3-7-58-s002.docx]

Table S2. Model descriptions and multi-model selection results for all models developed *a priori* to explain dispersal distance and probability of long-distance dispersal in juvenile Mt. Graham red squirrels (*Tamiasciurus hudsonicus grahamensis*) between 2010 and 2013. Models developed to test for dispersal hypotheses are indicated: DEN = local density, FRAG = habitat fragmentation, BEHAV = individual behaviour differences, CFR = competition for resources, IA = inbreeding avoidance.

| **Model name** | **Response = log dispersal distance, gaussian error structure** | | | | | | |  |  | |  |
| --- | --- | --- | --- | --- | --- | --- | --- | --- | --- | --- | --- |
| General Models Both Sexes | Dispersal Hypothesis | K | AICc | Delta AICc | AICc Wt. | Cum.Wt. | LL | Evidence Ratio | | |  |
| global |  | 13 | 89.43 | 0 | 0.99 | 0.99 | -24.13 |  |  | |  |
| intrinsic |  | 7 | 99.96 | 10.53 | 0.01 | 1 | -41.50 | 193.25 |  | |  |
| extrinsic |  | 8 | 113.55 | 24.13 | 0 | 1 | -46.98 |  |  | |  |
| null |  | 2 | 138.15 | 48.73 | 0 | 1 | -66.97 |  |  | |  |
| natalpatch |  | 4 | 142.11 | 52.68 | 0 | 1 | -66.69 |  |  | |  |
| Female Models |  | K | AICc | Delta AICc | AICc Wt. | Cum.Wt. | LL | Evidence Ratio | | |  |
| female.resources.locomotion | CFR, BEHAV | 5 | 35.82 | 0 | 0.82 | 0.82 | -9.91 |  |  | |  |
| female.resources | CFR | 4 | 39.98 | 4.16 | 0.1 | 0.93 | -14.56 | 8.01 |  | |  |
| female.resources*density | CFR, DEN | 5 | 43.41 | 7.59 | 0.02 | 0.95 | -14.94 |  |  | |  |
| female.resources.territories | CFR | 5 | 43.73 | 7.91 | 0.02 | 0.96 | -14.56 |  |  | |  |
| female.resources.behavior | CFR, BEHAV | 8 | 43.97 | 8.15 | 0.01 | 0.98 | -3.70 |  |  | |  |
| female.mother.mass | CFR | 3 | 44.24 | 8.43 | 0.01 | 0.99 | -18.49 |  |  | |  |
| female.locomotion | BEHAV | 3 | 45.21 | 9.39 | 0.01 | 0.99 | -18.94 |  |  | |  |
| intrinsic |  | 7 | 47.93 | 12.11 | 0 | 1 | -12.30 |  |  | |  |
| female.density | CFR, DEN | 3 | 48.25 | 12.43 | 0 | 1 | -20.58 |  |  | |  |
| female.resource.competition | CFR | 4 | 49.19 | 13.38 | 0 | 1 | -19.54 |  |  | |  |
| female.bci | CFR | 3 | 51.75 | 15.93 | 0 | 1 | -22.24 |  |  | |  |
| female.inbreeding | IA | 4 | 52.9 | 17.08 | 0 | 1 | -21.40 |  |  | |  |
| null |  | 2 | 53.75 | 17.93 | 0 | 1 | -24.62 |  |  | |  |
| extrinsic |  | 8 | 54.79 | 18.97 | 0 | 1 | -13.39 |  |  | |  |
| female.natalpatch | FRAG | 4 | 57.57 | 21.75 | 0 | 1 | -23.88 |  |  | |  |
| global |  | 13 | 189.81 | 154 | 0 | 1 | 9.09 |  |  | |  |
| Male Models |  | K | AICc | Delta AICc | AICc Wt. | Cum.Wt. | LL | Evidence Ratio | | |  |
| male.resources.locomotion | CFR, BEHAV | 5 | 56.22 | 0 | 0.82 | 0.82 | -21.23 |  |  | |  |
| male.locomotion | BEHAV | 3 | 59.66 | 3.44 | 0.15 | 0.97 | -26.31 | 5.58 |  | |  |
| male.resources.behavior | CFR, BEHAV | 8 | 65.34 | 9.12 | 0.01 | 0.98 | -19.13 |  |  | |  |
| male.resources | CFR | 4 | 65.48 | 9.27 | 0.01 | 0.99 | -27.79 |  |  | |  |
| intrinsic |  | 7 | 66.84 | 10.63 | 0 | 0.99 | -23.31 |  |  | |  |
| male.mother.mass | CFR | 3 | 66.86 | 10.64 | 0 | 1 | -29.93 |  |  | |  |
| male.resources.territories | CFR | 5 | 67.27 | 11.05 | 0 | 1 | -27.13 |  |  | |  |
| male.bci | CFR | 3 | 73.51 | 17.29 | 0 | 1 | -33.31 |  |  | |  |
| null |  | 2 | 74.77 | 18.55 | 0 | 1 | -35.18 |  |  | |  |
| male.inbreeding | IA | 4 | 77.87 | 21.65 | 0 | 1 | -34.19 |  |  | |  |
| male.competition | CFM | 5 | 79.12 | 22.9 | 0 | 1 | -33.40 |  |  | |  |
| male.natalpatch | FRAG | 4 | 79.69 | 23.47 | 0 | 1 | -35.13 |  |  | |  |
| extrinsic |  | 8 | 80.14 | 23.92 | 0 | 1 | -28.28 |  |  | |  |
| global |  | 13 | 99.48 | 43.26 | 0 | 1 | -13.99 |  |  | |  |
| **Response = binary long distance dispersal (> = 150 m males, > = 100 m females), binomial error structure** | | | | | | | | | |  | |
| General Models Both Sexes |  | K | AICc | Delta AICc | AICc Wt. | Cum.Wt. | LL | Evidence Ratio | | |  |
| global |  | 11 | 52.77 | 0 | 0.98 | 0.98 | -10.31 |  |  | |  |
| intrinsic |  | 6 | 61.05 | 8.27 | 0.02 | 1 | -23.45 | 62.61 |  | |  |
| extrinsic |  | 6 | 68.52 | 15.75 | 0 | 1 | -27.26 |  |  | |  |
| null |  | 1 | 82.83 | 30.06 | 0 | 1 | -40.38 |  |  | |  |
| natalpatch |  | 3 | 86.38 | 33.61 | 0 | 1 | -39.98 |  |  | |  |
| Female Models |  | K | AICc | Delta AICc | AICc Wt. | Cum.Wt. | LL | Evidence Ratio | | |  |
| female.resources.locomotion | CFR, BEHAV | 4 | 25.48 | 0 | 0.62 | 0.62 | -6.92 |  |  | |  |
| female.resources | CFR | 3 | 27.72 | 2.25 | 0.2 | 0.82 | -10.06 | 3.07 |  | |  |
| female.locomotion | BEHAV | 2 | 29.97 | 4.5 | 0.06 | 0.88 | -12.67 |  |  | |  |
| female.resources.territories | CFR | 4 | 30.98 | 5.5 | 0.04 | 0.92 | -10.06 |  |  | |  |
| female.resource.competition | CFR | 3 | 32.4 | 6.92 | 0.02 | 0.94 | -12.60 |  |  | |  |
| extrinsic |  | 7 | 33.04 | 7.56 | 0.01 | 0.95 | -5.21 |  |  | |  |
| female.bci | CFR | 2 | 33.21 | 7.74 | 0.01 | 0.97 | -14.31 |  |  | |  |
| female.mother.mass | CFR | 2 | 33.81 | 8.34 | 0.01 | 0.98 | -14.61 |  |  | |  |
| female.resources*density | CFR | 4 | 34.13 | 8.66 | 0.01 | 0.98 | -11.96 |  |  | |  |
| female.density | CFR, DEN | 2 | 34.21 | 8.74 | 0.01 | 0.99 | -14.85 |  |  | |  |
| female.inbreeding | IA | 3 | 35.42 | 9.94 | 0 | 1 | -14.11 |  |  | |  |
| null |  | 1 | 36.53 | 11.06 | 0 | 1 | -17.19 |  |  | |  |
| female.natalpatch | FRAG | 3 | 38.45 | 12.97 | 0 | 1 | -15.70 |  |  | |  |
| intrinsic |  | 6 | 39.96 | 14.49 | 0 | 1 | -10.75 |  |  | |  |
| female.resources.behavior | CFR, BEHAV | 7 | 41.62 | 16.15 | 0 | 1 | -6.81 |  |  | |  |
| global |  | 12 | 128 | 102.52 | 0 | 1 | 0.00 |  |  | |  |
| Male Models |  | K | AICc | Delta AICc | AICc Wt. | Cum.Wt | LL | Evidence Ratio | | |  |
| male.resources.locomotion | CFR, BEHAV | 4 | 31.39 | 0 | 0.74 | 0.74 | -10.52 |  |  | |  |
| male.locomotion | BEHAV | 2 | 33.6 | 2.21 | 0.24 | 0.98 | -14.55 | 3.02 |  | |  |
| male.resources | CFR | 3 | 41.43 | 10.04 | 0 | 0.99 | -17.17 |  |  | |  |
| male.mother.mass | CFR | 2 | 41.95 | 10.56 | 0 | 0.99 | -18.73 |  |  | |  |
| male.resources.behavior | CFR, BEHAV | 7 | 42.26 | 10.87 | 0 | 0.99 | -10.13 |  |  | |  |
| intrinsic |  | 6 | 42.51 | 11.12 | 0 | 1 | -13.05 |  |  | |  |
| male.resources.territories | CFR | 4 | 43.59 | 12.2 | 0 | 1 | -16.84 |  |  | |  |
| male.bci | CFR | 2 | 44.52 | 13.13 | 0 | 1 | -20.04 |  |  | |  |
| null |  | 1 | 45.39 | 14 | 0 | 1 | -21.63 |  |  | |  |
| male.inbreeding | IA | 3 | 48.81 | 17.43 | 0 | 1 | -20.98 |  |  | |  |
| male.competition | CFM | 4 | 48.85 | 17.46 | 0 | 1 | -19.68 |  |  | |  |
| male.natalpatch | FRAG | 3 | 49.55 | 18.16 | 0 | 1 | -21.36 |  |  | |  |
| extrinsic |  | 7 | 50.14 | 18.75 | 0 | 1 | -15.27 |  |  | |  |
| global |  | 12 | 58.67 | 27.28 | 0 | 1 | 0.00 |  |  | |  |
| **Model Name** | **Variables** |  |  |  |  |  |  |  |  | |  |
| global | mother.spring.mass, ppn.female, occ.mids.ha, occ.male.ha, occ.female.ha, bci, MIS2, MIS4, OF3, OF4, logpatch.area | | | | | | | | | |  |
| intrinsic | bci, MIS2, MIS4, OF3, OF4 | | |  |  |  |  |  |  | |  |
| extrinsic | mother.spring.mass, ppn.female, midocc.ha, occmale.ha, occfemale.ha, logpatch.area | | | | | | | |  | |  |
| null | intercept only |  |  |  |  |  |  |  |  | |  |
| bci | bci |  |  |  |  |  |  |  |  | |  |
| female.density | occ.female.ha |  |  |  |  |  |  |  |  | |  |
| female.inbreeding | ppn.male, occ.male.ha |  |  |  |  |  |  |  |  | |  |
| female.resource.competition | ppn.female, occ.female.ha | |  |  |  |  |  |  |  | |  |
| female.resources*density | mother.spring.mass, occ.female.ha, mother.spring.mass*occ.female.ha | | | | | | |  |  | |  |
| locomotion | OF4 |  |  |  |  |  |  |  |  | |  |
| male.inbreeding | ppn.female, occ.females.ha | |  |  |  |  |  |  |  | |  |
| mother.mass | mother.spring.mass |  |  |  |  |  |  |  |  | |  |
| natalpatch | logpatch.area, patch.code | |  |  |  |  |  |  |  | |  |
| resources | mother.spring.mass, bci | |  |  |  |  |  |  |  | |  |
| resources.behavior | mother.spring.mass, bci, MIS2, MIS4, OF3, OF4 | | | |  |  |  |  |  | |  |
| resources.locomotion | mother.spring.mass, bci, OF4 | | |  |  |  |  |  |  | |  |
| resources.territories | mother.spring.mass, occ.mids.ha, bci | | | |  |  |  |  |  | |  |
