# Supplementary material for: Altered natal dispersal at the range periphery: The role of behavior, resources, and maternal condition
Source: Ecol Evol. 2016 Nov 30;7(1):58–72. doi: 10.1002/ece3.2612 (PMC5216619; doi:10.1002/ece3.2612)
Supplement: Supplementary file 3 [file ECE3-7-58-s003.docx]

Table S3. Description of variables used in multi-model selection to explain dispersal distance and probability of long-distance dispersal in juvenile Mt. Graham red squirrels (*Tamiasciurus hudsonicus grahamensis*) between 2010 and 2013.

| **Model variables** | **Description** |
| --- | --- |
| **Intrinsic factors** |  |
| bci | body condition index: ( juvenile mass at time of capture (g) /shin length (mm)) / estimated days since birth) |
| MIS2 | PC 2 from MIS trials; positive weight for behaviours associated with alert vigilance and mirror contact |
| MIS4 | PC 4 from MIS trials; positive wieght for climbing or hanging from arena walls |
| OF3 | PC 3 from OF trials; positive weight for chewing and digging |
| OF4 | PC 4 from OF trials; positive weight for locomotion |
| **Extrinsic factors** |  |
| mother.spring.mass | body mass (g) of mother during spring (March - May) of birth year |
| occ.mids.ha | local density of occupied red squirrel middens within a 100 m radius buffer / 3.14 (100m^2 circle = 3.14 ha) |
| occ.male.ha | local density of red squirrel middens occupied by males within a 100 m radius buffer / 3.14 |
| occ.female.ha | local density of red squirrel middens occupied by females within a 100 m radius buffer / 3.14 |
| ppn.male | proportion of an individual's litter that are male |
| ppn.female | proportion of an individual's litter that is female |
| logpatch.area | log area (m^2) of an individual's natal patch |
| patch.code | code indicating whether the natal midden fell within the core of the natal patch (2) or at the margins (1) |
| **Global** | intrinsic + extrinsic |
| **Null** | intercept only |
