# Supplementary material for: Altered natal dispersal at the range periphery: The role of behavior, resources, and maternal condition
Source: Ecol Evol. 2016 Nov 30;7(1):58–72. doi: 10.1002/ece3.2612 (PMC5216619; doi:10.1002/ece3.2612)
Supplement: Supplementary file 4 [file ECE3-7-58-s004.docx]

Table S4. Natal dispersal distances and proportion of individuals dispersing in juvenile male and female Mt. Graham red squirrels (*Tamiasciurus hudsonicus grahamensis*) by year between 2010 and 2013.

| **Year** | **Males** | **Females** | **All** |
| --- | --- | --- | --- |
| **2010** |  |  |  |
| proportion dispersing | 0.57 | 0.25 | 0.40 |
| dispersal distance | 793.6 ± 921.3 (1.0 – 2,418.7) | 67.6 ± 34.2 (14.0 – 102.8) | 406.4 ± 710.6 (0.0 – 2,418.7) |
| **2011** |  |  |  |
| proportion dispersing | 1.00 | 0.67 | 0.85 |
| dispersal distance | 1,117.8 ± 1,635.7 (217.0 – 4,787.6) | 915.1 ± 1,170.8 (62.7 – 2,521.0) | 1,033.3 ± 1,403.1 (62.7 – 4,787.6) |
| **2012** |  |  |  |
| proportion dispersing | 0.42 | 0.50 | 0.44 |
| dispersal distance | 1,120.5 ± 1,337.1 (12.0 – 3,426.0) | 98.3 ± 28.1 (70.0 – 133.0) | 847.9 ± 1,223.2 (12.0 – 3,426.0) |
| **2013** |  |  |  |
| proportion dispersing | 0.63 | 0.22 | 0.41 |
| dispersal distance | 772.0 ± 907.6 (29.1 – 2,514.2) | 325.8 ± 826.7 (9.1 – 2,528.4) | 548.9 ± 872.9 (9.1 – 2,528.4) |
| **χ^2^_3 d.f._** proportion | 6.45 | 3.99 | 7.56 |
| ***p*** value proportion | 0.09 | 0.26 | 0.06 |
| **χ^2^_3 d.f._** distance | 0.97 | 5.71 | 5.98 |
| ***p*** value distance | 0.81 | 0.13 | 0.11 |
