# Supplementary material for: Altered natal dispersal at the range periphery: The role of behavior, resources, and maternal condition
Source: Ecol Evol. 2016 Nov 30;7(1):58–72. doi: 10.1002/ece3.2612 (PMC5216619; doi:10.1002/ece3.2612)
Supplement: Supplementary file 5 [file ECE3-7-58-s005.docx]

Appendix S1. Quantifying Natal patch size

To delineate patches of red squirrel habitat in the Pinaleños based on MGRS use, we compiled lifetime telemetry locations between September 2010 and February 2014 for individuals that were radio-collared as juveniles and identified thresholds for use in several forest structural characteristics derived from LiDAR remote sensing data (see Laes *et al.* 2009; Mitchell *et al.* 2012). We sampled seven 25-m resolution LiDAR-derived raster layers at 9,424 MGRS telemetry locations, and extracted values for percent canopy cover, mean tree height, standard deviation in tree height, total basal area, live basal area, slope, and elevation. We reclassified raster layers into 6 classes (0 – 5) based on the summary statistics derived from samples of each LiDAR layer: zero-minimum value = class 0, minimum to 0.5 of first quartile = class 1, 0.5 of first quartile to first quartile = class 2, first quartile to mean = class 3, mean to third quartile = class 4, and third quartile to maximum value used = class 5. This scale was reversed for slope since MGRS are associated with gentle slopes (Smith and Mannan 1994). We used the Weighted Overlay tool in ArcGIS to create a habitat suitability model (1 least suitable, 5 most suitable) where each output cell is the weighted mean of all reclassified layers. We followed Girvetz & Greco's (2007, 2009) Patch Morph algorithm in ArcGIS model builder to create habitat patches with quality and marginal edge habitat delineated. For quality patch interiors, we specified MGRS habitat as having habitat suitability model values ≥ 3, a conservative gap crossing threshold of 50 m (two pixels; see Bakker & Van Vuren 2004), and a 90% density filter, meaning that 90% of cells in a 50 m circular radius of a focal cell must have a value ≥ 3. For edge patches, we specified an 80% density filter, and added core and edge models to create a 2-zone patch model where 2 = quality patch interior and 1 = patch edge. Finally, we converted the 2-zone patches to polygons and calculated area in hectares for all patches (Figure 1). We extracted the patch area in hectares and patch code (quality patch interior or edge) associated with each individual’s natal and settlement location and used these as explanatory variables in subsequent natal dispersal models. Finally, we determined the distance to the nearest patch from each natal patch.

Bakker, V. J. and D. H. Van Vuren. 2004. Gap-crossing decisions by the red squirrel, a forest-dependent small mammal. Conservation Biology 18:689–697.

Girvetz, E. H. and S. E. Greco. 2007. How to define a patch: a spatial model for hierarchically delineating organism-specific habitat patches. Landscape Ecology 22:1131–1142.

Girvetz, E. H. and S. E. Greco. 2009. Multi-scale predictive habitat suitability modeling based on hierarchically delineated patches: an example for yellow-billed cuckoos nesting in riparian forests, California, USA. Landscape Ecology 24:1315–1329.

Laes, D., T. Mellin, C. Wilcox, J. Anhold, P. Maus, D. A. Falk, et al. 2009. Mapping vegetation structure in the Pinaleño Mountains using lidar. RSAC-0118-RPT1. U.S. Department of Agriculture, Forest Service, Remote Sensing Applications Center. 84 p. Salt Lake City, UT.

Mitchell, B., M. Walterman, T. Mellin, C. Wilcox, A. M. Lynch, J. Anhold, et al. 2012. Mapping vegetation structure in the Pinaleño Mountains using lidar - phase 3: forest inventory and modeling. RSAC-10007-RPT1. U.S. Department of Agriculture, Forest Service, Remote Sensing Applications Center. 17 p. Salt Lake City, UT.

Smith, A. A. and R. W. Mannan. 1994. Distinguishing characteristics of Mount Graham red squirrel midden sites. The Journal of Wildlife Management 58:437–445.
